# Step away from depression—results from a multicenter randomized clinical trial with a pedometer intervention during and after inpatient treatment of depression

**DOI:** 10.1007/s00406-023-01646-2

**Published:** 2023-08-17

**Authors:** Julia Große, Charlotte Huppertz, Astrid Röh, Viola Oertel, Sara Andresen, Niklas Schade, Franziska Goerke-Arndt, Anna Kastinger, Nikola Schoofs, Philipp Arthur Thomann, Karsten Henkel, Berend Malchow, Jens Plag, Aleksandra Terziska, Ralf Brand, Frank Helmig, Alexander Schorb, Dirk Wedekind, Maria Jockers-Scherübl, Frank Schneider, Moritz Bruno Petzold, Andreas Ströhle

**Affiliations:** 1 Klinik für Psychiatrie und Psychotherapie, Campus Charité Mitte, Charité—Universitätsmedizin Berlin, Corporate member of Freie Universität Berlin, Humboldt‐Universität zu Berlin and Berlin Institute of Health, Berlin, Germany; 2 Department of Psychiatry, Psychotherapy and Psychosomatics, Faculty of Medicine, RWTH Aachen University, Aachen, Germany; 3 Department of Psychiatry, Psychotherapy and Psychosomatics of the University Augsburg, Bezirkskrankenhaus Augsburg, Medical Faculty, University of Augsburg, Augsburg, Germany; 4 Klinik für Psychiatrie, Psychosomatik und Psychotherapie, Universitätsklinikum Frankfurt/Main, Frankfurt am Main, Germany; 5 Fachklinik für Psychiatrie, Psychosomatik und Psychotherapie Flensburg der DIAKO NF, Flensburg, Germany; 6 Department of Psychiatry and Psychotherapy, University Medical Center, Göttingen, Germany; 7 Department of Psychiatry and Psychotherapy, University Medical Center Hamburg-Eppendorf (UKE), Hamburg, Germany; 8 Department of Psychiatry and Psychotherapy, Oberhavel Kliniken GmbH, Hennigsdorf, Germany; 9 Psychotherapy and Psychosomatics, University Hospital of Psychiatry, Paracelsus Medical University, Salzburg, Austria; 10 Center for Mental Health, Odenwald District Healthcare Center, Erbach, Germany; 11 Sport and Exercise Psychology, University of Potsdam, Potsdam, Germany; 12 University Hospital, Heinrich-Heine-University Düsseldorf, Düsseldorf, Germany; 13 Department of Psychology, Medical School Berlin, Berlin, Germany

**Keywords:** Depression, Physical activity, Steps, Inpatient treatment, Accelerometer, Psychotherapy

## Abstract

Evidence for the effectiveness of physical activity (PA) in the treatment of depression prevails for outpatients with mild and moderate symptom levels. For inpatient treatment of severe depression, evidence-based effectiveness exists only for structured and supervised group PA interventions. The Step Away from Depression (SAD) study investigated the effectiveness of an individual pedometer intervention (PI) combined with an activity diary added to inpatient treatment as usual (TAU). In this multicenter randomized controlled trial, 192 patients were randomized to TAU or TAU plus PI. The two primary outcomes at discharge were depression—blindly rated with the Montgomery–Åsberg Depression Rating Scale (MADRS)—and average number of daily steps measured by accelerometers. Secondary outcomes were self-rated depression and PA, anxiety, remission and response rates. Multivariate analysis of variance (MANOVA) revealed no significant difference between both groups for depression and daily steps. Mean MADRS scores at baseline were 29.5 (SD = 8.3) for PI + TAU and 28.8 (SD = 8.1) for TAU and 16.4 (SD = 10.3) and 17.2 (SD = 9.9) at discharge, respectively. Daily steps rose from 6285 (SD = 2321) for PI + TAU and 6182 (SD = 2290) for TAU to 7248 (SD = 2939) and 7325 (SD = 3357). No differences emerged between groups in secondary outcomes. For severely depressed inpatients, a PI without supervision or further psychological interventions is not effective. Monitoring, social reinforcement and motivational strategies should be incorporated in PA interventions for this population to reach effectiveness.

**Supplementary Information:**

The online version contains supplementary material available at 10.1007/s00406-023-01646-2.

## Introduction

Depression has a tremendous burden on people’s health [1]. It is one of the most lethal mental diseases with a 1.51 to 1.90 higher death risk (Hazard’s Ratio) for patients compared to healthy persons [2–4]. Furthermore, severe additional health risks like stroke or cardiovascular diseases are associated with depression [5, 6]. Research shows several causes for these risks—a generally less healthy life style, with worse nutrition and less physical activity, as well as (neuro-) biological and neuroendocrinological processes [7–12].

Treatment costs for depression are the highest compared to all other mental diseases [13, 14]. Psychotherapy and antidepressant medication represent the main treatment options [15, 16]. More severe depressive disorders go along with more expensive treatments—inpatient treatment accounts for almost half of the direct treatment costs [17]. The search for both—higher clinical effectiveness and lower costs of the treatment of depression—is an urgent task in mental health care. Our trial is meant to contribute to that matter.

Physical activity already plays an important role in the prevention of depression [18–22]. Even more strongly, as part of antidepressant treatment, it is increasingly recommended during last years [15, 16, 23]. The effectiveness of physical activity in the treatment of depression was shown in many reviews and confirmed in several meta-analyses [24–29]. Besides, results of no effectiveness have been equally reported [30], leaving questions about contradicting findings. A number of interventions have been developed and implemented [31, 32]. However, most studies and interventions focus on mild to moderate depression symptom level and on outpatient treatment [29, 33]. Little work has been done for patients with severe depression that undergo inpatient treatment. Thus, we are interested in new findings concerning PA interventions for this relevant population.

Furthermore, we want to include the aspect of integrated health care because it helps lowering costs and makes treatment more efficient [34–36]. Therefore, we look for interventions that offer the chance of being continued on the long term after inpatient treatment in an outpatient setting [37] and that can be supported by non-specialized health care personnel. An easy and inexpensive method for increasing PA is the use of pedometers combined with the formulation of step goals [38]. Pedometers can be easily used by patients and provide the potential of cross-sectional use for both inpatient and outpatient treatment. While 10.000 steps per day were originally propagated by a Japanese company to promote commercial pedometers, research shows that already 7000 steps have beneficial effects on health [39]. Daily step counts lower than 4000 are associated with higher mortality [40].”

To our knowledge, no study so far investigated the add-on-effect of a pedometer intervention for inpatients with depression. In this trial, we examine the effectiveness of a pedometer intervention combined with the use of an activity book as an add-on-intervention for the inpatient treatment of severe depression.

### Methods

## Trial design and overview

This multicenter, longitudinal, randomized controlled parallel-group trial was conducted in ten psychiatric clinics in Germany and Austria: *Charité Universitätsmedizin Berlin*, *Alexianer St. Hedwig Hospital*, *University Hospital RWTH Aachen*, *DIAKO Hospital Flensburg*, *Hospital LMU Munich*, *University Hospital Frankfurt*, *University Hospital Göttingen*, *University Hospital Salzburg*, *Oberhavel Clinic Hennigsdorf* and *Health Center Odenwaldkreis*. The study was registered as a clinical trial (see Supplementary Materials S1, ClinicalTrials.gov Identifier: NCT02850341, registered January 08, 2016). A detailed study protocol was published containing the description of methodological and statistical aspects of the study [41]. Treatment as usual (TAU) plus a pedometer intervention (PI) was compared to TAU only at baseline (t0) and at discharge (t1b). If t1b data were missing, we replaced it by t1a data (4 weeks after hospital admission) as last observation carried forward. Points of measurement altogether were day 1–3 after hospital admission (baseline, t0), after 4 weeks (t1a) (if treatment length extended 4 weeks), at discharge (t1b), and 6 months after hospital admission (follow-up). Enrollment took place from August 2016 until January 2020.

## Study population and randomization

Patients were included with an age between 18 and 65 years and with major depression as primary diagnosis. Psychiatrists in each clinic in charge of the patient performed the clinical diagnosis by taking the medical history, using additional questionnaires and preexisting clinical health records of the patient. In addition, a planned treatment length of 4 weeks was needed. Exclusion diagnoses were psychotic depression, borderline personality disorder, schizophrenia, anorexia nervosa, current substance addiction and dementia. Further reasons for exclusion were medical risks or inability to walk at least 5000 steps, current use of a pedometer or other activity tracker, as well as a baseline level of more than 10.000 steps per day. Patients were randomized in a 1:1 ratio stratified by center using a computerized random number generator. Randomization remained concealed until the end of the baseline measurement.

## Study procedure

Investigators in all centers were clinical staff and/or advanced students of psychology or medicine, all trained in standard conduction of the trial and of the assessments. Patients underwent baseline measurements within the first three working days of hospital treatment. They filled out questionnaires online via SoSci-Survey [42] or, in the occurrence of any technical or personal constraints, by paper–pencil. At baseline only, we collected sociodemographic variables, number of former depressive episodes and treatments, somatic diseases, medication and physiological parameters (blood pressure, heart rate, body weight, blood glucose, and laboratory values of triglycerides, cholesterol, high-density lipoprotein and low density lipoprotein). All other measures were collected at every measure point (see Supplementary Materials S2). TAU consisted of inpatient psychotherapy (group and/or individual), pharmacotherapy, adjuvant sociotherapy, occupational therapy, music therapy, physiotherapy and/or exercise therapy. Patients of the TAU plus PI received an Omron Walking style IV pedometer (Model HJ-325-EW) and an activity diary. Originating from the patient’s initially blinded number of steps as baseline measurement, they were instructed to raise their daily number of steps by 500 for the coming week. If the goal of 500 additional daily steps was fulfilled on at least four days of one week and 10.000 steps had not been reached yet, the goal for step count should be raised again by 500 daily steps for the next week. Patients had to fill in their daily steps into the diary throughout the whole hospital treatment and continuing afterward for altogether 6 months maximum.

## Outcome measures

Our two primary outcomes were the MADRS sum score and average daily step count at discharge. Trained raters blind to the group assignment of the patient conducted the MADRS interview. Trainer’s interrater correlations showed high reliability with Cronbach’s Alpha of 0.978 (CI: 0.95–0.99) [41]. Step count was assessed with ActiGraph GT1M accelerometers (ActiGraph) [43–45]. Patients wore the accelerometers around their waist for three consecutive days during wake time. The outcome variable was mean daily step count (valid if at least two days with at least 10 h of wear time).

As secondary outcomes we used the International Physical Activity Questionnaire (IPAQ) [46], the Beck Depression Inventory II (BDI-II) [47] and the Beck Anxiety Inventory (BAI) [48]. Exploratory, we collected nine further measures for post hoc analyses: (1) psychopathological symptoms, (2) health-related quality of life, (3) self-reported depressive symptoms, for physical activity: (4) self-efficacy, (5) intention, (6) self-concordance of goal-striving, (7) outcome expectations, (8) planning and barrier planning and (9) general self-efficacy. In addition, we collected data concerning the adherence to and the evaluation of the intervention, treatment history, critical life events during the follow-up time interval and adverse events during the trial (see Supplementary Materials S2).

## Sample size and power calculation

Based on other add-on exercise studies [30, 49–51], we assumed a small to moderate effect size for both differences in MADRS sum score and mean step count. Results of these trials showed an absolute mean difference of 4 points in the MADRS [50]. A difference of 1.6–1.9 in the MADRS score represents a minimal clinically meaningful change [52]. A difference of 4 points on the MADRS is associated with reduction of 0.5 in the CGI [53]. We therefore estimated a mean difference of four points in the MADRS score as a substantially meaningful clinical difference between treatment groups. Pedometer studies showed an increase of approximately 1000 steps per day in the intervention group [51, 54]. 1000 steps more per day are associated with essential improvements for individual health [39]. Thus, we expected a number of 1000 steps as a mean difference between groups. For reaching a power of 95% in a parallel group, fixed sample trial with multivariate analysis of variance (MANOVA), 264 cases were necessary according to G*Power Version 3.2.1). Taking into account a loss rate of 34% for dropout and missing data, we aimed at an overall sample size of 400 patients.

## Statistical analysis

In our primary analysis, we analyzed patients with complete data. In our second full analysis set, data of all eligible and randomized patients were analyzed. For this set, missing data of the two outcome variables MADRS and mean daily steps were imputed with multiple imputations (number of imputations: 10; relative efficiency: 94–96%). The multiple imputation model was based on age, sex, hospitalization length, body weight and the following variables, respectively, for baseline, t1a, t1b and follow-up: step count, MADRS, BDI-II, time in light, moderate, vigorous, very vigorous PA (tresholds of Freedson [55], using ActiLife Software [56]), sedentary time, IPAQ and BAI. All data were analyzed using SPSS Statistics 25. Significance levels were set to *p* = 0.05.

First, we calculated descriptive statistics for the sociodemographic and clinical data of patients. Remission and response rates were calculated, as well as percentages of patients meeting the WHO recommendations for PA [57].

As specified in our study protocol, the two primary outcomes depression and steps were likely to correlate [58] and we planned a multivariate analysis of variance (MANOVA) for them as dependent variables with group as independent variable. However, our results showed no significant correlation between depression and steps. Furthermore, both dependent variables were not normally distributed (showing significant results in the Kolmogorov-Smirnoff-Test). Homogeneity was given (Box’s *M* = 9.96, *p* = 0.18). According to Finch et al. [59], under these conditions MANOVA is still more powerful than nonparametric tests so we continued the analysis with MANOVA. Secondary outcomes of groups were compared using ANOVA. Differences in frequencies between groups were calculated using exact Fisher’s test (if only two variables were compared) or Pearson’s Chi-square test. We used Bonferroni correction to adjust for multiple testing.

## Results

### Participant characteristics and missing data

**Fig. 1:**
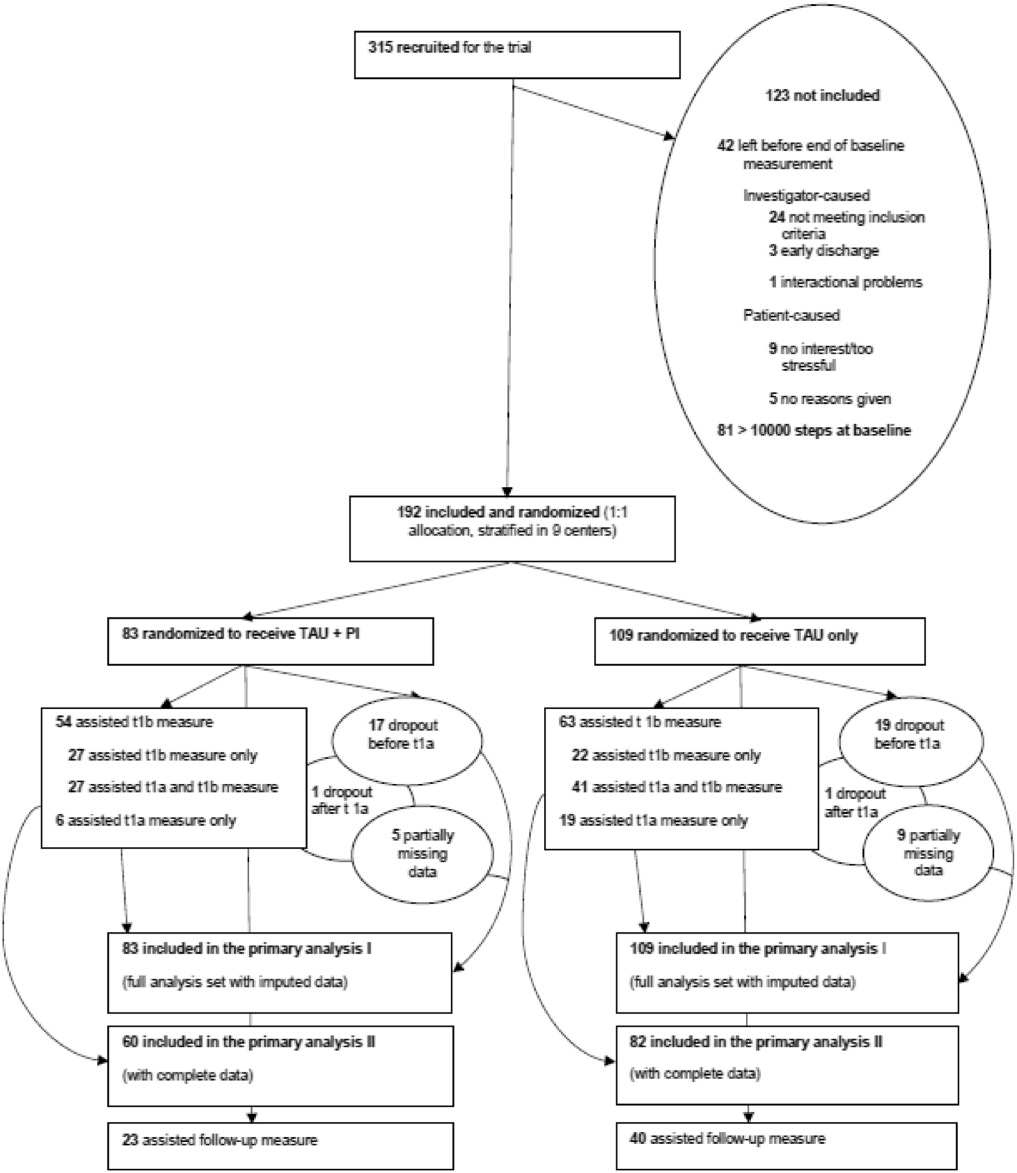
Flowchart. *TAU*—treatment as usual; *PI*—pedometer intervention; *T1b*, at discharge; T1a, after four weeks

Altogether, 732 eligible patients were asked to participate. 315 of them consented, leading to an acceptance rate of 43.03 percent. However, enrollment took place from July 2016 to January 2020, for limits in staff and resources impeded an further extension of the trial. Figure 1 shows the flow diagram with additional information about non-participants and dropouts. Our final sample size to analyze was *N* = 192 (*N* = 83 TAU plus PI, *N* = 109 TAU), providing a power of 0.86 for a parallel group, fixed sample trial with multivariate analysis of variance (MANOVA) with *p* = 0.05 (using G*Power Version 3.1.9.4).

Of 732 eligible persons asked, 315 consented to participate, Patients excluded or refusing to participate after baseline measurement had an age, sex, educational level, MADRS score or daily step count that was comparable to those participating (see Supplementary Materials S3a and S3b).

Sixty (72%) of the TAU + PI and 82 (75%) of the TAU participants with complete data could be used in the primary analysis. Trial sites showed significantly different rates of cases with complete data, ranging from 41.67 to 100.00%, *χ*^2^ = 21.4, df = 9, *p* = 0.01 (with the constraint of a 30% cell frequency < 5).

22% of the participants in the TAU + PI and 18% in the TAU group dropped out of the study until discharge. 14 (7.2%) patients gave no reasons for their quitting of the trial, 12 (6.2%) did lose their interest in the study. 10 (5.2%) were discharged earlier than 4 weeks after baseline. One patient (0.5%) was injured and one patient (0.5%) felt overtaxed by the trial. Dropout patients had a significant lower BDI-II score at baseline (mean [SD] BDI-II score, 26.3 [12.3]) than completers (mean [SD] BDI-II score, 32.0 [10.6]; *t* = 2.86, df = 183, *p* = 0.005). No significant differences emerged for trial site, treatment condition, age, sex, educational level, steps per day, MADRS and IPAQ.

Participants completing the full study protocol had missing data of 6.02% of the TAU + PI and 8.26% of the TAU group for the primary outcomes. For the full analysis set with all 192 cases, we included complete cases and imputed cases of dropout and missing data.

Baseline group characteristics are shown in Table 1***.*** Groups were comparable regarding most variables including age, sex and the primary outcomes. Overall, patients were 41.7 years old (SD = 13.3), 106 (55%) were women, 98 (52%) had a vocational and 47 (25%) an academic educational level. 74 patients (39%) were seeking work and 84 (44%) were (self-)employed. Mean MADRS score at baseline was 29 (SD = 8.3) and mean steps per day were 6222 (SD = 2300). Mean treatment length was 49 days (Median = 43; SD = 27) with no difference between groups.

**Table 1:** Baseline demographics and characteristics

Characteristic	TAU + PI ^(*n* = 83^)	TAU ^(*n* = 109)^
Age, mean (SD)	42.0 (13.5) [*n* = 77]	41.6 (13.3) [*n* = 101]
	No. (%)	
Sex		
Female	48 (58)	58 (53)
Male	32 (39)	46 (42)
Divers	1 (1.2)	0 (0)
Not provided	2 (2.4)	5 (4.6)
Educational level		
None	14 (17)	19 (17)
Vocational training	38 (46)	60 (55)
University degree	24 (19)	23 (21)
Not provided	7 (8.4)	7 (6.4)
Employment status		
Seeking work	36 (43)	38 (35)
Undergoing training	9 (11)	10 (9)
(Self-)Employed	31 (37)	53 (49)
Not provided	7 (8.4)	8 (7.3)
Main diagnose		
F32.1	12 (15)	9 (8)
F32.2	8 (10)	21 (19)
F33.1	20 (24)	18 (17)
F33.2	43 (52)	61 (56)
No. of comorbid diagnoses		
1	10 (12)	20 (18)
2	5 (6.0)	3 (2.8)
> 2	2 (2.4)	2 (1.8)
	Mean (SD)	
MADRS score	29.48 (8.26) [*n* = 75]	28.8 (8.1) [*n* = 101]
Steps per day	6257.61 (2321.35) [*n* = 80]	6182.60 (2290.48) [*n* = 71]
BDI-II	29.61 (10.33) [*n* = 80]	31.83 (11.75) [n = 105]
IPAQ total MET minutes per week	2819.42 (3202.91) [*n* = 72]	2812.01 (3651.27) [*n* = 97]
IPAQ sedentary MET minutes per week	453.94 (246.37) [*n* = 79]	459.01 (265.55) [*n* = 106]
Number of depressive episodes before	4.76 (4.89) [*n* = 76]	3.64 (3.84) [*n* = 97]
Body mass index	26.49 (6.59) [*n* = 68]	26.69 (5.79) [*n* = 91]

*TAU*—treatment as usual.* PI*—pedometer intervention. *MADRS*—Montgomery–Åsberg Depression Rating Scale. *BDI*−II—Beck Depression Inventory II. *IPAQ*—International Physical Activity Questionnaire. *MET*—metabolic equivalent of task

Differences in sociodemographic, clinical and outcome variables between the different trial centers were marginal and are presented in detail in Supplementary Materials S4.

### Primary outcomes

Mean MADRS score at discharge was 16.4 (SD = 10.3) in the TAU + PI and 17.2 (SD = 9.9) in the TAU group. Mean steps per day were 7248 (SD = 2939) and 7325 (SD = 3357), respectively (see Table 2).

**Table 2:** Primary and main secondary outcomes

	Mean (SD)			
Outcomes	TAU + PI	TAU	Absolute difference (95% CI)	*p*
MADRS score	16.42 (10.30) [*n* = 57]	17.21 (9.90) [*n* = 76]	– 0.79 (– 4.28 to 2.70)	0.375
Steps per day	7248.46 (2939.02) [*n* = 42]	7324.72 (3357.15) [*n* = 52]	– 76.27 (– 1385.56 to 1233.02)	0.457
BDI-II	19.39 (11.86) [*n* = 60]	20.94 (13,29) [*n* = 81]	– 1,56 (– 5.83 to 2.72)	0.564
IPAQ Total MET	4200.87 (4539.00) [*n* = 60]	4213.89 (5891.25) [*n* = 80]	– 13.03 (– 1821.37 to 1795.32)	0.996
IPAQ Sedentary time	330.57 (165.69) (*n* = 58]	367.65 (177.17) [n = 81]	– 37.09 (– 95.76 to 21.59)	0.207
BAI	15.79 (11.63) [*n* = 58]	15.19 (11.24) [*n* = 80]	0,61 (– 3.28 to 4.50)	0.865

*TAU*—treatment as usual. *PI*—pedometer intervention.* CI*—confidence interval. *MADRS*—Montgomery–Åsberg Depression Rating Scale. *BDI*−II—Beck Depression Inventory II. *IPAQ*—International Physical Activity Questionnaire. *MET*—metabolic equivalent of task. *BAI*—Beck Anxiety Inventory

**Fig. 2:**
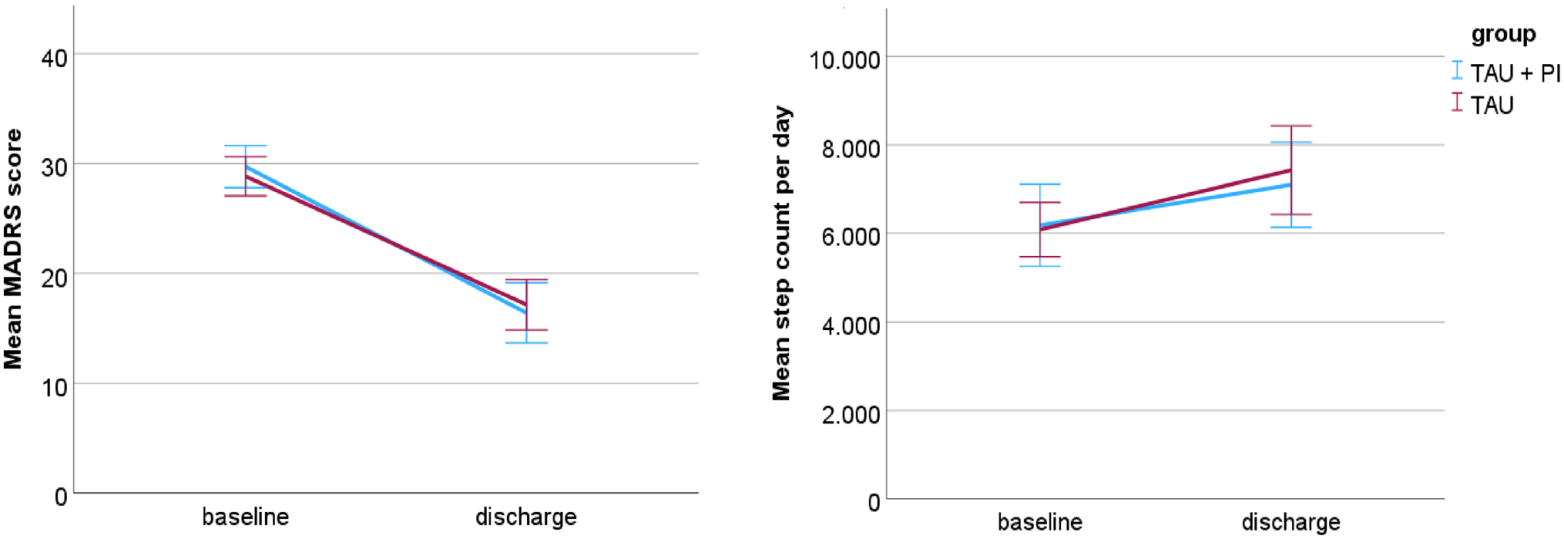
Primary outcomes Montgomery–Åsberg Depression Rating Scale (MADRS) and mean step count per day at baseline and at discharge. Error bars show 95% confidence intervals. *TAU* treatment as usual. *PI*—pedometer intervention

The correlation between the two primary outcomes was not significant (*rs*(85) = − 0.12, *p* = 0.25). Figure 2 presents the comparison of the two treatment groups concerning means and standard deviations for the primary outcomes at baseline and discharge.

Our MANOVA in the primary analysis II set showed no significant difference between groups for both dependent variables (F (2, 84) = 0.67, *p* = 0.52 (Pillai’s trace *V* = 0.16)). Likewise, in the imputed primary analysis I set, MANOVA in all of the 10 data sets of the multiple imputation showed no significant difference between treatments (see Supplementary Materials S5).

No differences emerged in response and remission rates. Forty-six percent of the TAU + PI group and 40% in the TAU group had more than 50% reduction in the MADRS from baseline to discharge (see Supplementary Materials S6). Remission rate was 25% (14/57) for the TAU + PI and 18% (14/72) for the TAU group.

The percentage of participants in each group meeting criteria of WHO-recommended PA at discharge was higher when self-rated. Measured with accelerometers, 44% of the TAU + PI and 43% of the TAU group met WHO recommendations. For the self-rated IPAQ, these were 58% and 51%, respectively.

### Secondary outcomes

Groups showed no significant differences in means for both treatment groups at discharge for IPAQ, BDI-II and BAI. In the IPAQ, total weekly physical activity (estimated by weighting time spent in each activity intensity with its estimated metabolic equivalent task (MET) energy expenditure) of the TAU + PI group was 4200.87 MET minutes per week (SD = 4539.00). The TAU group showed 4213.89 MET minutes per week (SD = 5891.25). The means of the BDI-II were 19.39 (SD = 11.86) and 20.94 (SD = 13.29), those of the BAI 15.79 (SD = 11.63) and 15.19 (SD = 11.24), respectively. Further results of secondary outcomes are presented in Supplementary Materials S7.

### Additional exploratory analyses

Likewise, no outcome differences emerged between groups in exploratory analyses for subgroups of patients with different sex, younger or older patients or patients with moderate versus severe depression according to MADRS cut-off value of 35 points. However, for severely depressed patients only, the correlation between steps per day and depression at baseline was significant, *r*(25) = − 0.61, *p* < 0.001 (see Supplementary Materials S8e). For moderately depressed patients, this correlation remained non-significant. Furthermore, treatment effect was not different between responders and non-responders.

Besides, we explored the amount of PA on different weekdays at baseline. Patients showed least steps on Sundays (5375.92, SD = 2617.95) and Mondays (5678.71, SD = 2892.30) and most steps on Tuesdays (7269.32, SD = 2389.30) and Saturdays (6800.33, SD = 3204.88). Detailed information on all exploratory results can be found in Supplementary Materials S8.

### Adverse events, compliance and acceptance of the intervention

No serious adverse events were reported throughout the study course.

**Fig. 3:**
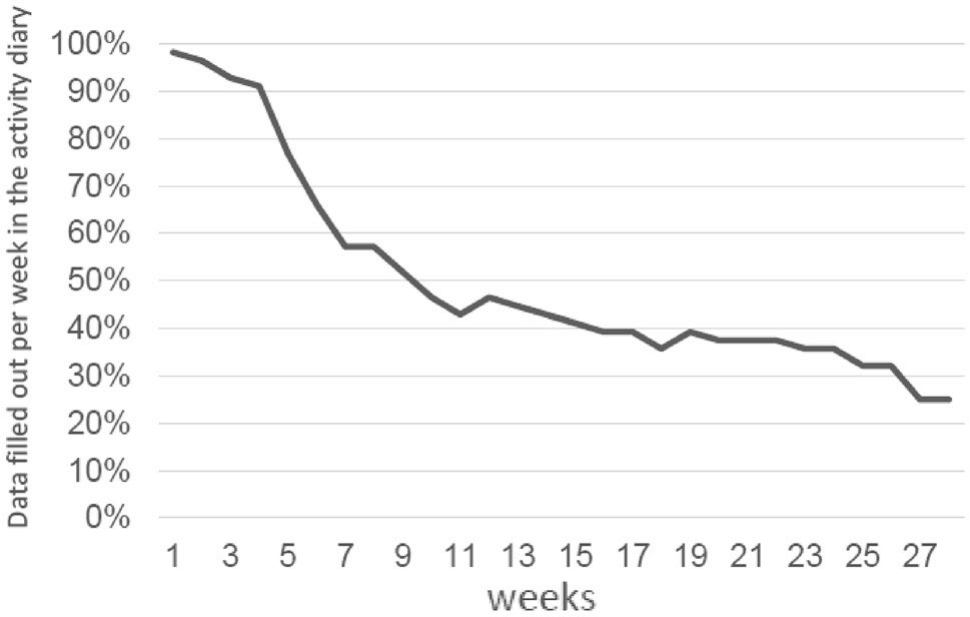
Percentage of data filled out per week in the activity diary in the course of time (*n* = 56)

We received copies of the activity book of 56 participants (93% of TAU + PI participants of T1 measurements). Compliance with the PI decreased in the course of treatment. Figure 3 shows compliance rates over all 24 weeks starting with 98.2% of patients using the activity book in the first, 91.1% in the fourth, 57.1% in the 8th and 25% in the 28th week. Those participants who kept the diary did so with diminishing completeness: Whereas in the first week, patients filled out 95.1% of the activity book, this percentage declined to 93.4% in the second, 87.8% in the third and 80.9% in the fourth week.

Full information about results of the program evaluation can be found in Supplementary Materials S9 and S10. The PI was rated as moderately helpful by participants with a mean score of 4.1 (SD = 1.3, *N* = 69) on a 1 (“not helpful at all”) to 6 (“very helpful”) Likert scale. Of 69 participants answering the evaluation questionnaire, 42 (61%) said that they were more physically active because of the PI. Using grades of 1 (very good) to 6 (insufficient) participants evaluated the PI with 2.1 (SD = 0.9). Main openly formulated positive evaluations of the PI were that it was “motivating” (13/35, 37%), that “monitoring helped” (8/35, 23%) and that it gave more control about the amount of physical activity performed (4/35, 11%). As disadvantages participants mentioned the “accelerometer measurement” (5/35, 14%), “nothing” (4/35, 11%) and that “steps were too biased as a measure for PA” (3/35, 9%). As possible improvements “the use of a fitness wristband” (3/35, 9%) and “more support” (2/35, 6%) were suggested.

## Discussion

This multicenter trial, to our knowledge, was the first to investigate the effect of an add-on pedometer intervention in the inpatient treatment of depression. Results showed no significant differences between treatment groups. Neither depressive symptoms, nor step level were different between patients with additional PI and patients undergoing TAU only. Equally, we found no differences in secondary outcome measures between groups. This was unexpected, for research has shown that PA has a significant effect in the treatment of depression [24, 25].

One reason for our results could be the comparatively high step levels of our sample at baseline. This contradicts decreased PA of depressive patients generally observed in other studies [60, 61]. However, this is in line with findings that inpatients move more than outpatients [62] and that pedometer (or accelerometer) wearing leads to an increase in PA [38]. These high baseline values could have diminished the chance to measure between-group-differences. For further research projects in naturalistic settings, routine measurement of PA for every patient should be integrated into inpatient treatment so that this effect can be reduced as much as possible. Moreover, using an armwrist tool may attract less attention and lead to a more reliable measurement of PA [63]. All the more, continuous measuring with armwrist tools can provide the possibility of measuring heart rate, heart rate variability and rest and sleep times.

A second reason could be that compliance and adherence rates of patients decreased over weeks. Thus, our intervention may not have been successfully implemented in the course of treatment. If adherence is ensured by participation of inpatients in structured physical exercise like aerobic sessions, studies show that PA as an add-on to TAU significantly helps decreasing depression [64, 65]. We do not know how effective the PI would be when thoroughly adhered to by patients. Adherence problems were reported already in other PA trials with depressive patients [66, 67]. Therefore, our results are in line with other researchers suggesting that adherence to PA interventions should be supported by behavior change interventions [28, 68–71]. For instance, developing concrete action plans, barrier management and working with situational cues might be useful adherence-increasing strategies to be implemented in PA interventions. Furthermore, positive affective reactions while performing PA need to be supported—PA interventions should meet these requirements, e.g., by paying attention to positive environment and fun rather than pressure. Finally, regular monitoring and reward for the patients if keeping up with PA should be included.

Third, also patients of the TAU condition could have tried to make more steps during treatment because they were motivated by the mere presence of the conducted trial in the psychiatric ward. It could not be hidden that the aim of the PI was to increase PA and this was known to TAU patients. Additionally, it cannot be ruled out that our study attracted patients more strongly motivated to increase their PA than those not participating which could have biased results.

Our dropout rates were as expected and comparable to other exercise (as well as psychotherapy) studies for depression [72, 73]. Therefore, we can assume that the study design as well as the conduct of the trial were of good quality and did not bias results.

Interestingly, the correlation between depression and steps was not statistically significant. This was contrary to our expectations as other studies showed significant correlations between depression and PA [58]. It is possible that in our specific population of depressive inpatients the general relationship between these two variables is different from that for outpatients. Notably, another recent study showed a significant negative correlation between depression and PA for outpatients but not for inpatients [74]. In our subgroup analysis, we found a significant negative correlation only for patients with severe depression. Thus, it might be that the correlation between PA and depression might only be significant for patients with either light or severe depression but may be less distinct for patients with moderate depression. Further research is needed to assess this question.

Moreover, the measurement of steps could have constraints with regard to its reliability. Admission as well as discharge weeks of treatment might be different from usual PA of depressive patients. Moreover, we recorded steps for three consecutive days. Also, we did not differentiate between days in the week and on the weekend. Results show that patients move less on Sundays and Mondays. This might be a specific finding for inpatient treatment. Sundays have already been shown to be associated with less PA, in general. Reduction in PA on Mondays in psychiatric wards could be specifically due to more waiting time for visits of the attending doctors or to more routine examinations. Hence, these differences of PA for different weekdays should be taken into account for further research in an inpatient population.

On the one hand, maybe a period of 5 or 7 days would have led to other estimates of daily step level. On the other hand, adherence rates would then possibly be reduced due to a higher burden. However, three days of monitoring were shown to be sufficient for reliable and valid estimates of PA, especially, if the sample is large enough [75–77].

Alternative explanations for our results may also be that exercise is not causally connected with decrease in depression [30], that personality traits may be a non-considered variable [78] or that the role of genes needs to be regarded, too [79, 80]. In addition, individual profit as well as contraindication might have happened that cannot be shown by focusing on means of data but would tribute individualized statistical methods.

In our study, the sample fulfilled the WHO recommendations for PA as much as patients with severe mental diseases [62] and—as expected—lower compared to healthy samples [81]. Rates of self-rated measures were lower. This confirms the discrepancy between subjective and objective measurement of PA and the need to integrate both types of measurement in exercise research [41]. Some studies showed weak validity of the IPAQ [82] and using other self-report questionnaires for PA with higher psychometric quality such as the simple physical activity questionnaire (SIMPAQ) [83] may be of advantage.

To sum up, although participants subjectively evaluated the intervention as helpful, our results implicate that, in inpatient treatment, the use of a pedometer and an activity book alone does not lead to an additional significant change of depression or step level on the long term. This is in line with studies showing that patients need more support to maintain motivation and to develop volitional strategies [84, 85]. Regular supervision of the program would be promising [23, 68] and patients of our study made this wish (see Supplementary Materials S10). Additionally, emotional reactions [86] and smartphone use as a tool option [87] should be considered.

### Limitations and strengths

We examined a simple non-expensive PA intervention with an excellent balance of potential benefits and risks in a naturalistic setting of inpatient treatment for depressive patients in a multicenter design. We measured depression as well as PA with two measures, respectively, including blinded clinical rating of depression severity accelerometry-based PA, and we provided long-term data during and follow-up data after the release from hospital.

Restrained power was a limitation of our trial due to recruitment of only 315 instead of planned 400 subjects as well as substantial loss of data. Even having used multiple imputation for the analysis I, power still provided a 14% chance of not detecting a true effect. However, power was sufficiently strong to detect a small effect in primary outcomes. For the analysis II with complete data cases, results would have been underpowered with 0.74. Therefore, we cannot totally rule out the possibility of a type II error not detecting an effect.

Furthermore, contribution of several trial sites to the final study sample was unequal. However, we found no differences in baseline values or results when analyzing data center-wise. Double-blindness was not possible and acceptance rate was low. This could have contributed to a bias in results. Finally, the high baseline PA of patients was unexpected. Selection of participants might have been biased in the way that patients with no interest in PA could have preferred not to participate in the study. This could have reduced the chance of finding an effect of our intervention for depressive inpatients with low PA. Future studies should pay attention to attract especially patients with low PA for the inclusion of PA trials.

## Conclusion

We found that adding a PI to TAU without further supervision or psychological interventions does not lead to less depression severity or more steps at release from hospital of depressive inpatients. This can be due to high effectiveness of inpatient treatment in general and low adherence for the PI. As a conclusion, adherence to PA interventions should be supported by regular supervision. Future research work in this field should plan, describe and implement adherence-supporting strategies when using PA interventions for patients with depression.

### Supplementary Information

Below is the link to the electronic supplementary material.

Supplementary file1 (PDF 2160 KB)

## Data Availability

The data of this study and analyses presented in the paper are available upon request from first author.
