# Supplementary material for: Step away from depression—results from a multicenter randomized clinical trial with a pedometer intervention during and after inpatient treatment of depression
Source: Eur Arch Psychiatry Clin Neurosci. 2023 Aug 17;274(3):709–21. doi: 10.1007/s00406-023-01646-2 (PMC10995038; doi:10.1007/s00406-023-01646-2)
Supplement: Supplementary file 1 — Supplementary file1 (PDF 2160 KB) [file 406_2023_1646_MOESM1_ESM.pdf]

Supplement Material

Step Away from Depression—Results from a multicenter randomized clinical trial with a pedometer intervention during and after inpatient treatment of depression

European Archives of Psychiatry and Clinical Neuroscience

Julia Große[1]; Charlotte Huppertz[2]; Astrid Röh[3]; Viola Oertel[4]; Sara Andresen[5]; Niklas Schade[6,7]; Franziska Goerke-Arndt[8]; Anna Kastinger[9]; Nikola Schoofs[1]; Philipp Arthur Thomann [10]; Karsten Henkel[2]; Berend Malchow[6]; Jens Plag[1]; Aleksandra Terziska[1]; Ralf Brand[11]; Frank Helmig[5]; Alexander Schorb[9]; Dirk Wedekind[6]; Maria Jockers-Scherübl[8]; Frank Schneider[12]; Moritz Bruno Petzold[1]; Andreas Ströhle[1]

[1]Klinik für Psychiatrie und Psychotherapie, Campus Charité Mitte, Charité—Universitätsmedizin Berlin, corporate member of Freie Universität Berlin, Humboldt-Universität zu Berlin, and Berlin Institute of Health, Berlin Germany
[2] Department of Psychiatry, Psychotherapy and Psychosomatics, Faculty of Medicine, RWTH Aachen University, Aachen, Germany
[3] Department of Psychiatry, Psychotherapy and Psychosomatics of the University Augsburg, Bezirkskrankenhaus Augsburg, Medical Faculty, University of Augsburg, Augsburg, Germany
[4] Klinik für Psychiatrie, Psychosomatik und Psychotherapie, Universitätsklinikum Frankfurt/Main
[5] Fachklinik für Psychiatrie, Psychosomatik und Psychotherapie Flensburg der DIAKO NF
[6] Department of Psychiatry and Psychotherapy, University Medical Center, Göttingen, Germany
[7] Department of Psychiatry and Psychotherapy, University Medical Center Hamburg-Eppendorf (UKE), Hamburg, Germany
[8] Oberhavel Kliniken GmbH, Department of Psychiatry and Psychotherapy, Hennigsdorf, Germany
[9] University Hospital of Psychiatry, Psychotherapy and Psychosomatics, Paracelsus Medical University, Salzburg, Austria
[10] Center for Mental Health, Odenwald District Healthcare Center, Erbach, Germany
[11] University of Potsdam, Sport and Exercise Psychology, Potsdam, Germany
[12] University Hospital, Heinrich-Heine-University Düsseldorf

Author for correspondence: Julia Große, Charité – Universitätsmedizin Berlin, corporate member of Freie Universität Berlin and Humboldt Universität zu Berlin, Department of Psychiatry and Neurosciences, Charitéplatz 1, 10117 Berlin, Germany.
E-mail address: julia.grosse@charite.de
https://orcid.org/0000-0002-4488-0033

S1: Registration of the SAD study in clinicaltrials.gov

https://clinicaltrials.gov/ct2/show/NCT02850341

Tracking Information	
**First Submitted Date** ICMJE	July 22, 2016
**First Posted Date** ICMJE	August 1, 2016
**Last Update Posted Date**	September 1, 2021
**Actual Study Start Date** ICMJE	July 2016
**Actual Primary Completion Date**	January 2020 (Final data collection date for primary outcome measure)
**Current Primary Outcome Measures** ICMJE **(submitted: July 27, 2016)**	• Change in average number of steps per day [Time Frame: From baseline (1.-3. day of inpatient treatment) to end of inpatient treatment (3 days before end of inpatient treatment)]<br>Number of steps per day is objectively measured using accelerometry (ActiGraph GT1M)<br>• Change in depressive symptoms - clinician rated [Time Frame: From baseline (1.-3. day of inpatient treatment) to end of inpatient treatment (3 days before end of inpatient treatment)]<br>Depressive symptoms are rated by a blind study employee using Montgomery–Åsberg Depression Rating Scale (MADRS)
**Original Primary Outcome Measures** ICMJE	*Same as current*
**Change History**	Complete list of historical versions of study NCT02850341 on ClinicalTrials.gov Archive Site
**Current Secondary Outcome Measures** ICMJE **(submitted: July 27, 2016)**	• Change in physical activity - subjective [Time Frame: From baseline (1.-3. day of inpatient treatment) to end of inpatient treatment (3 days before end of inpatient treatment)]<br>Physical activity is subjectively measured with the International Physical Activity Questionnaire (IPAQ)<br>• Change in physical activity - objective [Time Frame: From baseline (1.-3. day of inpatient treatment) to end of inpatient treatment (3 days before end of inpatient treatment)]<br>Physical activity is objectively measured using accelerometry (ActiGraph GT1M)<br>• Change in psychopathological symptoms [Time Frame: From baseline (1.-3. day of inpatient treatment) to end of inpatient treatment (3 days before end of inpatient treatment)]<br>Psychopathological symptoms are measured by the Symptom Checklist 27 (SCL-27)<br>• Change in health-related quality of life [Time Frame: From baseline (1.-3. day of inpatient treatment) to end of inpatient treatment (3 days before end of inpatient treatment)]

	Health-related quality of life is measured using the Short-Form Health Questionnaire (SF-12)<br>• Change in depressive symptoms [Time Frame: From baseline (1.-3. day of inpatient treatment) to end of inpatient treatment (3 days before end of inpatient treatment)]<br>Depressive symptoms are measured using the Beck Depression Inventory (BDI II)<br>• Change in anxiety symptoms [Time Frame: From baseline (1.-3. day of inpatient treatment) to end of inpatient treatment (3 days before end of inpatient treatment)]<br>Anxiety symptoms are measured using the Beck Anxiety Inventory (BAI)<br>• Change in self-efficacy for physical activity [Time Frame: From baseline (1.-3. day of inpatient treatment) to end of inpatient treatment (3 days before end of inpatient treatment)]<br>Self-efficacy for physical activity is measured using six likert scaled items by Scholz, U., Sniehotta, F. & Schwarzer, R. (2005)<br>• Change in intention for physical activity [Time Frame: From baseline (1.-3. day of inpatient treatment) to end of inpatient treatment (3 days before end of inpatient treatment)]<br>Intention for physical activity is measured using two likert scaled items by Sniehotta, F. F., Schwarzer, R., Scholz, U., & Schüz, B. (2005)<br>• Change in self concordance of the motivation for physical activity [Time Frame: From baseline (1.-3. day of inpatient treatment) to end of inpatient treatment (3 days before end of inpatient treatment)]<br>Self concordance of the Motivation for physical activity is measured using the "Sport- und bewegungsbezogene Selbstkonkordanz Skala", which consists of 12 likert scaled items<br>• Change in outcome expectancies for physical activity [Time Frame: From baseline (1.-3. day of inpatient treatment) to end of inpatient treatment (3 days before end of inpatient treatment)]<br>Outcome expectancies for physical activity are measured using 6 likert scaled items by Lippke S., Ziegelmann, J. P. & Schwarzer, R. (2005)<br>• Change in planning and barrier planning for physical activity [Time Frame: From baseline (1.-3. day of inpatient treatment) to end of inpatient treatment (3 days before end of inpatient treatment)]<br>Planning and barrier planning for physical activity is measured using 8 likert scaled items by Sniehotta, F. F. et al. (2005) / Renner, B. (2007)<br>• Duration of inpatient treatment (Number of days) [Time Frame: Duration of inpatient treatment is assessed at the end of inpatient treatment]<br>• Change in general self-efficacy [Time Frame: From baseline (1.-3. day of inpatient treatment) to end of inpatient treatment (3 days before end of inpatient treatment)]<br>General self-efficacy is measured using the General-Self-Efficacy Scale (GSE)
**Original Secondary Outcome Measures** ICMJE	*Same as current*
**Current Other Pre-specified Outcome Measures**	*Not Provided*
**Original Other Pre-specified Outcome Measures**	*Not Provided*
**Descriptive Information**	
**Brief Title** ICMJE	Step Away From Depression - Evaluation of a Pedometer Intervention With Inpatients With Major Depression

**Official Title** ICMJE	Step Away From Depression - Evaluation of a Pedometer Intervention With Inpatients With Major Depression - A Randomized Controlled Trial
**Brief Summary**	The purpose of this study is to determine if the use of pedometers can help depressive inpatients in psychiatric clinics to increase their level of physical activity. Therefore patients are given a pedometer and instructions how to raise their level of daily steps. Intervention group is compared with a control group that is receiving treatment-as-usual. The intervention is hypothesized to increase number of daily steps and have positive effects on mood, depression and anxiety.
**Detailed Description**	*Not Provided*
**Study Type** ICMJE	Interventional
**Study Phase** ICMJE	Not Applicable
**Study Design** ICMJE	Allocation: Randomized<br>Intervention Model: Parallel Assignment<br>Masking: None (Open Label)<br>Primary Purpose: Supportive Care
**Condition** ICMJE	Depression
**Intervention** ICMJE	Device: Pedometer<br>Patients receive a pedometer with instructions how to raise their number of daily steps
**Study Arms** ICMJE	• Experimental: Intervention group<br>Patients receive a pedometer and instructions how to raise their physical activity<br>Intervention: Device: Pedometer<br>• No Intervention: Control group<br>Patients receive treatment-as-usual
**Publications ***	*Not Provided*

*** Includes publications given by the data provider as well as publications identified by ClinicalTrials.gov Identifier (NCT Number) in Medline.**

Recruitment Information

**Recruitment Status** ICMJE	Completed
**Actual Enrollment** ICMJE **(submitted: August 27, 2021)**	292
**Original Estimated Enrollment** ICMJE **(submitted: July 27, 2016)**	400
**Actual Study Completion Date** ICMJE	January 2020
**Actual Primary Completion Date**	January 2020 (Final data collection date for primary outcome measure)
**Eligibility Criteria** ICMJE	Inclusion Criteria:<br>Major Depression, moderate to severe as main diagnosis<br>Inpatient status<br>Prospected inpatient treatment of at least 4 weeks<br>Able to understand german<br>Exclusion Criteria:

	Physical disease or disability that makes it impossible to reach the goal 5000 steps per day<br>Borderline personality disorder<br>Bipolar Disorder<br>Schizophrenia<br>Anorexia Nervosa<br>Dementia<br>Psychotic Depression<br>Pregnancy<br>Acute suicidality<br>Substance dependancies with actual consumption (except nicotine)<br>Current pedometer-use<br>More than 10.000 steps per day
**Sex/Gender** ICMJE	Sexes Eligible for Study: All
**Ages** ICMJE	18 Years to 65 Years (Adult, Older Adult)
**Accepts Healthy Volunteers** ICMJE	No
**Contacts** ICMJE	*Contact information is only displayed when the study is recruiting subjects*
**Listed Location Countries** ICMJE	Austria, Germany
**Removed Location Countries**	
**Administrative Information**	
**NCT Number** ICMJE	NCT02850341
**Other Study ID Numbers** ICMJE	SAD
**Has Data Monitoring Committee**	No
**U.S. FDA-regulated Product**	*Not Provided*
**IPD Sharing Statement** ICMJE	Plan to Share IPD: No
**Responsible Party**	Prof. Dr. Andreas Ströhle, Charite University, Berlin, Germany
**Study Sponsor** ICMJE	Charite University, Berlin, Germany
**Collaborators** ICMJE	Robert-Enke-Stiftung
**Investigators** ICMJE	Principal Investigator: Andreas Ströhle, Prof.Dr. Charité Universitätsmedizin Berlin - Klinik für Psychiatrie und Psychotherapie
**PRS Account**	Charite University, Berlin, Germany
**Verification Date**	August 2021

S2: Time table of measurements

		t0 (baseline)	t1a (4 weeks)	t1b (discharge)	t2 (follow-up)
	Physical Activity (number of items)				
Objective	Accelerometer measurement for 3 days (Primary Outcome)	x	x	x	x
Subjective	**Q:** International Physical Activity Questionnaire (IPAQ) short version (7)	x	x	x	x
Adherence	**AD:** Copy of step protocols (IG only)		x	x	x
	Depression (number of items)				
Objective	**I:** Montgomery-Åsberg Depression Rating Scale (Primary Outcome) (10)	x	x	x	x
Subjective	**Q:** Beck-Depression-Inventory II (21)	x	x	x	x
	General, physical and clinical measures (number of items)				
General measures	**Q:** sociodemographic data, medication	x			
Physiological measures	**HR:** blood pressure, heart rate, body weight and blood glucose	x			
Laboratory values	**HR:** tryglycerides, cholesterol, HDL and LDL	x			
Clinical History	**Q:** Anamnestic Questions (6)	x			
Psychiatric Symptoms	**Q:** Symptom Checklist 27 (27)	x	x	x	x
Anxiety	**Q:** Beck Anxiety Inventory (21)	x	x	x	x
General Health Quality	**Q:** Short-Form Health-Survey 12 (12)	x	x	x	x
Intervention evaluation	**Q:** Questions about the implementation of PI (5)				x
Treatment history	**Q:** Concomitant treatment since discharge (13)				x
Critical life events	**Q:** Social readjustment rating scale (43)				x
	Components of the Motivation Volition Model (number of items)				
Intention, motivation and volition	**Q:** Intention (2), Physical activity specific self-efficacy (6), Outcome expectancies (6), Self Concordance Scale (12), Action planning (4), Barrier management (4)	x	x	x	x

S3a: Comparison participants vs. non-participants in the study for categorical variables

			Participating in the study	Not participating in the study	*p (Pearson Chi-Square)*
Study center [n = 292]	Berlin	Count	71	6	
		% within study center	92,2%	7,8%	
	München	Count	35	2	
		% within study center	94,6%	5,4%	
	Flensburg	Count	28	0	
		% within study center	100,0%	0,0%	
	Aachen	Count	40	4	
		% within study center	90,9%	9,1%	
	Göttingen	Count	12	0	
		% within study center	100,0%	0,0%	
	Frankfurt	Count	30	3	
		% within study center	90,9%	9,1%	
	Salzburg	Count	4	2	
		% within study center	66,7%	33,3%	
	Hedwig	Count	32	1	
		% within study center	97,0%	3,0%	
	Erbach	Count	9	1	
		% within study center	90,0%	10,0%	
	Hennigsdorf	Count	11	1	
		% within study center	91,7%	8,3%	
	Total	Count	272	20	,25
		% within study center	93,2%	6,8%	
Sex [n = 276]	Female	Count	135	11	
		% within sex	92,5%	7,5%	
	Male	Count	121	6	
		% within sex	95,3%	4,7%	
	Divers	Count	3	0	
		% within sex	100,0%	0,0%	
	Total	Count	259	17	,57
		% within sex	93,8%	6,2%	
Educational level [n = 273]	Vocational training	Count	127	7	
		% within educational level	94,8%	5,2%	
	Technical college	Count	28	1	
		% within educational level	96,6%	3,4%	
	University degree	Count	54	7	
		% within educational level	88,5%	11,5%	
	none	Count	47	2	
		% within educational level	95,9%	4,1%	
	Total	Count	256	17	,87
		% within educational level	93,8%	6,2%	
Group [n = 289]	TAU + PI	Count	131	10	
		% within group	92,9%	7,1%	
	TAU	Count	141	7	
		% within group	95,3%	4,7%	
	Total	Count	272	17	,39
		% within group	94,1%	5,9%	

TAU, treatment as usual; PI, pedometer intervention.

S3b: Comparison participants vs. non-participants for metric variables

Group Statistics

	Participating	N	Mean	Std. Deviation	Std. Error Mean	*p* (t-test for equality of means)
MADRS Score baseline	Yes	252	29,06	8,250	,520	,729
	No	14	29,86	9,726	2,599	
Steps per day baseline	Yes	188	8897,7819	4283,74271	312,42405	,524
	No	10	8012,2000	4017,20598	1270,35207	
Age in years	Yes	258	41,78	13,090	,815	,953
	No	17	41,59	12,535	3,040	

MADRS, Montgomery–Åsberg Depression Rating Scale.

S4: Differences between trial centers

Differences between trial centers were calculated for: treatment length, age, number of school years, number of physical illnesses, BMI; baseline and discharge respectively: MADRS, daily steps, BDI, IPAQ Total MET, BAI.

**ANOVA revealed overall significant differences (omnibus test) in**	**Significant post hoc differences (with Bonferroni correction) emerged in**
treatment length (p = .044) with the highest in Frankfurt (60.58 days, SD = 33.02) and the lowest in Erbach (28.86 days, SD = 6.04).	**x**
number of school years (p = .042) with lowest 9.38 (SD = .74) in Erbach and highest in Salzburg (12.50, SD = .71).	**x**
daily steps at baseline (p = .034) with lowest 4266.00 (SD = 4393.01) in Salzburg and highest in Flensburg (8626.07, SD = 1308.28).	Daily steps at baseline between Flensburg and Erbach as well as Frankfurt and between Erbach and Flensburg.
IPAQ MET at baseline with lowest 1840.73 (SD = 1993.37) in Berlin and highest 6485.57 (SD = 7729.85) in Erbach.	**x**
MADRS at discharge with lowest 7.00 (SD = 2.83) in Erbach and highest 22.8 (SD = 6.50) in Göttingen.	MADRS at discharge with 7.00 (SD = 2.83) in Erbach and 22.8 (SD = 6.50) in Göttingen.
BDI at discharge with lowest 12.60 (SD = 13.74) in Erbach and highest 26.83 (SD = 12.66) in Berlin.	BDI at discharge between Berlin and Flensburg as well as Aachen.
BAI at discharge with lowest 8.95 (SD = 6.33) in Aachen and 22.00 (SD = 15.56) in Salzburg.	BAI at discharge between Berlin and Aachen.

MADRS, Montgomery–Åsberg Depression Rating Scale; BDI-II, Beck Depression Inventory II; IPAQ, International Physical Activity Questionnaire; MET, metabolic equivalent of task; BAI, Beck Anxiety Inventory.

S5: MANOVA results with multiple imputation data

Results of multivariate Tests

Imputation_		Value	F	Hypothesis df	Error df	Sig.
0	Pillai's Trace	,013	,374^{a}	2,000	56,000	,690
	Wilk's Lambda	,987	,374^{a}	2,000	56,000	,690
	Hotelling's Trace	,013	,374^{a}	2,000	56,000	,690
	Roy's Largest Root	,013	,374^{a}	2,000	56,000	,690
1	Pillai's Trace	,003	,248^{a}	2,000	189,000	,780
	Wilk's Lambda	,997	,248^{a}	2,000	189,000	,780
	Hotelling's Trace	,003	,248^{a}	2,000	189,000	,780
	Roy's Largest Root	,003	,248^{a}	2,000	189,000	,780
2	Pillai's Trace	,001	,096^{a}	2,000	189,000	,908
	Wilk's Lambda	,999	,096^{a}	2,000	189,000	,908
	Hotelling's Trace	,001	,096^{a}	2,000	189,000	,908
	Roy's Largest Root	,001	,096^{a}	2,000	189,000	,908
3	Pillai's Trace	,024	2,331^{a}	2,000	189,000	,100
	Wilk's Lambda	,976	2,331^{a}	2,000	189,000	,100
	Hotelling's Trace	,025	2,331^{a}	2,000	189,000	,100
	Roy's Largest Root	,025	2,331^{a}	2,000	189,000	,100
4	Pillai's Trace	,001	,068^{a}	2,000	189,000	,934
	Wilk's Lambda	,999	,068^{a}	2,000	189,000	,934
	Hotelling's Trace	,001	,068^{a}	2,000	189,000	,934
	Roy's Largest Root	,001	,068^{a}	2,000	189,000	,934
5	Pillai's Trace	,000	,027^{a}	2,000	189,000	,974
	Wilk's Lambda	1,000	,027^{a}	2,000	189,000	,974
	Hotelling's Trace	,000	,027^{a}	2,000	189,000	,974
	Roy's Largest Root	,000	,027^{a}	2,000	189,000	,974
6	Pillai's Trace	,010	,973^{a}	2,000	189,000	,380
	Wilk's Lambda	,990	,973^{a}	2,000	189,000	,380
	Hotelling's Trace	,010	,973^{a}	2,000	189,000	,380
	Roy's Largest Root	,010	,973^{a}	2,000	189,000	,380
7	Pillai's Trace	,004	,394^{a}	2,000	189,000	,675
	Wilk's Lambda	,996	,394^{a}	2,000	189,000	,675
	Hotelling's Trace	,004	,394^{a}	2,000	189,000	,675
	Roy's Largest Root	,004	,394^{a}	2,000	189,000	,675
8	Pillai's Trace	,004	,400^{a}	2,000	189,000	,671
	Wilk's Lambda	,996	,400^{a}	2,000	189,000	,671
	Hotelling's Trace	,004	,400^{a}	2,000	189,000	,671
	Roy's Largest Root	,004	,400^{a}	2,000	189,000	,671
9	Pillai's Trace	,001	,112^{a}	2,000	189,000	,894
	Wilk's Lambda	,999	,112^{a}	2,000	189,000	,894

	Hotelling's Trace	,001	,112[a]	2,000	189,000	,894
	Roy's Largest Root	,001	,112[a]	2,000	189,000	,894
10	Pillai's Trace	,003	,318[a]	2,000	189,000	,728
	Wilk's Lambda	,997	,318[a]	2,000	189,000	,728
	Hotelling's Trace	,003	,318[a]	2,000	189,000	,728
	Roy's Largest Root	,003	,318[a]	2,000	189,000	,728

a. Exact statistic

S6. Response and remission rates for MADRS at discharge

	Response (>50% MADRS reduction)	Remission (MADRS Score <8)
	**No. (%)**	**No. (%)**
TAU + PI (n = 57)	26 (46)	14 (25)
TAU (n=72)	30 (40)	14 (18)
Total	56 (42)	28 (21)

MADRS, Montgomery–Åsberg Depression Rating Scale; TAU, treatment as usual; PI, pedometer intervention.

S7: Secondary outcomes at baseline and discharge

Secondary outcomes	Time point	Treatment as usual + Pedometer Intervention (n = 83)	Treatment as usual (n = 109)	*p* (t-test for equality of means)
		Mean (Standard Deviation)	Mean (Standard Deviation)	
Accelerometer-measured MVPA	baseline	30.73 (18.63) [n = 55]	31.32 (19.98) [n = 76]	0.866
	discharge	38.75 (22.87) [n = 45]	40.42 (27.45) [n = 60]	0.741
IPAQ total MET score	baseline	2819.42 (3202.91) [n = 72]	2812.01 (3651.27) [n = 97]	0.989
	discharge	4200.87 (4539.00) [n = 60]	4213.89 (5891.25) [n = 80]	0.989
IPAQ Sedentary MET score	baseline	453.94 (246.37) [n = 79]	459.01 (265.55) [n = 106]	0.895
	discharge	330.57 (165.69) [n = 60]	367.65 (177.18) [n = 80]	0.213
BDI-II score	baseline	29.61 (10.33) [n = 80]	31.83 (11.75) [n =105]	0.181
	discharge	19.37 (11.84) [n = 60]	21.19 (13.16) [n = 80]	0.399
BAI score	baseline	24.61 (11.57) [n = 80]	21.53 (12.02) [n =106]	0.080
	discharge	15.79 (11.63) [n = 58]	15.19 (11.24) [n = 80]	0.759
SCL-27 Global severity index	baseline	1.69 (0.71) [n = 78]	1.79 (0.74) [n =102]	0.340
	discharge	1.15 (0.76) [n = 59]	1.13 (0.74) [n = 81]	0.892
SF-12 Physical Component Summary	baseline	39.21 (10.26) [n = 72]	39.94 (10.73) [n = 105]	0.649
	discharge	45.80 (9.51) [n = 59]	42.99 (9.71) [n = 81]	0.875
SF-12	baseline	26.90 (7.67) [n = 72]	27.09 (8.19) [n = 105]	0.218

Mental Component Summary	discharge	36.62 (11.87) [n = 59]	38.59 (10.85) [n = 81]	0.537
General Self Efficacy	baseline	22.69 (5.20) [n = 77]	22.07 (6.27) [n =105]	0.479
	discharge	24.68 (6.18) [n = 60]	25.00 (5.69) [n = 81]	0.753
Specific self-efficacy for physical activity	baseline	2.71 (0.64) [n = 77]	2.55 (0.71) [n =106]	0.132
	discharge	2.86 (0.60) [n = 60]	2.86 (0.53) [n = 81]	0.960
Intention	baseline	3.13 (0.71) [n = 77]	3.06 (0.81) [n =106]	0.515
	discharge	3.33 (0.68) [n = 60]	3.28 (0.58) [n = 81]	0.658
Positive Outcome expectancy	baseline	3.32 (0.60) [n = 77]	3.20 (0.66) [n =106]	0.223
	discharge	3.33 (0.55) [n = 60]	3.33 (0.58) [n = 81]	0.933
Negative Outcome expectancy	baseline	2.02 (0.64) [n = 76]	1.87 (0.58) [n =105]	0.107
	discharge	1.79 (0.46) [n = 60]	1.79 (0.56) [n = 80]	0.975
Action planning	baseline	2.46 (0.92) [n = 81]	2.30 (0.93) [n =106]	0.263
	discharge	2.73 (0.79) [n = 59]	2.79 (0.70) [n = 81]	0.653
Barrier management	baseline	2.07 (0.70) [n = 81]	1.86 (0.77) [n =106]	0.057
	discharge	2.34 (0.70) [n = 59]	2.37 (0.72) [n = 81]	0.771
Self-concordance index	baseline	1.20 (1.72) [n = 80]	1.20 (1.52) [n =105]	0.998
	discharge	2.29 (1.75) [n = 50]	2.24 (1.56) [n = 62]	0.873
Days of treatment		48.30 (27.14) [n = 81]	48.86 (26.13) [n = 100]	0.887

MVPA, Minutes of moderate and vigorous physical activity per day. IPAQ, International Physical Activity Questionnaire; MET, Metabolic Equivalent of Task; BDI-II, Beck Depression Inventory II; BAI, Beck Anxiety Inventory; SCL-27, Symptom Checklist 27; SF-12, Short Form 12

S8: Exploratory analyses

S8a Subgroup analysis for symptom severity

Multivariate Tests[a]

MADRS_severity	Effect		Value	F	Hypothesis df	Error df	Sig.
moderate	Intercept	Pillai's Trace	,903	283,081[b]	2,000	61,000	<,001
		Wilks' Lambda	,097	283,081[b]	2,000	61,000	<,001
		Hotelling's Trace	9,281	283,081[b]	2,000	61,000	<,001
		Roy's Largest Root	9,281	283,081[b]	2,000	61,000	<,001
	Gruppe	Pillai's Trace	,040	1,287[b]	2,000	61,000	,283
		Wilks' Lambda	,960	1,287[b]	2,000	61,000	,283
		Hotelling's Trace	,042	1,287[b]	2,000	61,000	,283
		Roy's Largest Root	,042	1,287[b]	2,000	61,000	,283
severe	Intercept	Pillai's Trace	,900	94,169[b]	2,000	21,000	<,001
		Wilks' Lambda	,100	94,169[b]	2,000	21,000	<,001
		Hotelling's Trace	8,969	94,169[b]	2,000	21,000	<,001
		Roy's Largest Root	8,969	94,169[b]	2,000	21,000	<,001
	Gruppe	Pillai's Trace	,003	,035[b]	2,000	21,000	,966
		Wilks' Lambda	,997	,035[b]	2,000	21,000	,966
		Hotelling's Trace	,003	,035[b]	2,000	21,000	,966
		Roy's Largest Root	,003	,035[b]	2,000	21,000	,966

a. Design: Intercept + Gruppe

b. Exact statistic

Tests of Between-Subjects Effects

MADRS_severity	Source	Dependent Variable	Type III Sum of Squares	df	Mean Square	F	Sig.
moderate	Corrected Model	MADRS_discharge	196,651[a]	1	196,651	2,148	,148
		MittelSchritte_discharge	2118000,741[b]	1	2118000,741	,253	,617
	Intercept	MADRS_discharge	15051,901	1	15051,901	164,427	<,001
		MittelSchritte_discharge	2888746565,6	1	2888746565,6	345,351	<,001
	Gruppe	MADRS_discharge	196,651	1	196,651	2,148	,148
		MittelSchritte_discharge	2118000,741	1	2118000,741	,253	,617
	Error	MADRS_discharge	5675,584	62	91,542		
		MittelSchritte_discharge	518609052,77	62	8364662,142		
	Total	MADRS_discharge	22481,000	64			
		MittelSchritte_discharge	3590699178,9	64			
	Corrected Total	MADRS_discharge	5872,234	63			
		MittelSchritte_discharge	520727053,51	63			
severe	Corrected Model	MADRS_discharge	9,643[c]	1	9,643	,070	,794
		MittelSchritte_discharge	69,144[d]	1	69,144	,000	,998
	Intercept	MADRS_discharge	8742,976	1	8742,976	63,088	<,001
		MittelSchritte_discharge	1527457187,8	1	1527457187,8	93,667	<,001
	Gruppe	MADRS_discharge	9,643	1	9,643	,070	,794
		MittelSchritte_discharge	69,144	1	69,144	,000	,998
	Error	MADRS_discharge	3048,857	22	138,584		
		MittelSchritte_discharge	358760625,85	22	16307301,175		
	Total	MADRS_discharge	11952,000	24			
		MittelSchritte_discharge	1929748095,6	24			
	Corrected Total	MADRS_discharge	3058,500	23			
		MittelSchritte_discharge	358760695,00	23			

a. R Squared = ,033 (Adjusted R Squared = ,018)

b. R Squared = ,004 (Adjusted R Squared = -,012)

c. R Squared = ,003 (Adjusted R Squared = -,042)

d. R Squared = ,000 (Adjusted R Squared = -,045)

S8b Subgroup analysis for age lower or higher than 44 years (mean of age)

Multivariate Tests[a]

age_category	Effect		Value	F	Hypothesis df	Error df	Sig.
.	Intercept	Pillai's Trace	,905	9,481[b]	1,000	1,000	,200
		Wilks' Lambda	,095	9,481[b]	1,000	1,000	,200
		Hotelling's Trace	9,481	9,481[b]	1,000	1,000	,200
		Roy's Largest Root	9,481	9,481[b]	1,000	1,000	,200
	Gruppe	Pillai's Trace	,481	,926[b]	1,000	1,000	,512
		Wilks' Lambda	,519	,926[b]	1,000	1,000	,512
		Hotelling's Trace	,926	,926[b]	1,000	1,000	,512
		Roy's Largest Root	,926	,926[b]	1,000	1,000	,512
younger than 44	Intercept	Pillai's Trace	,902	193,077[b]	2,000	42,000	<,001
		Wilks' Lambda	,098	193,077[b]	2,000	42,000	<,001
		Hotelling's Trace	9,194	193,077[b]	2,000	42,000	<,001
		Roy's Largest Root	9,194	193,077[b]	2,000	42,000	<,001
	Gruppe	Pillai's Trace	,001	,023[b]	2,000	42,000	,977
		Wilks' Lambda	,999	,023[b]	2,000	42,000	,977
		Hotelling's Trace	,001	,023[b]	2,000	42,000	,977
		Roy's Largest Root	,001	,023[b]	2,000	42,000	,977
older than 43	Intercept	Pillai's Trace	,901	168,001[b]	2,000	37,000	<,001
		Wilks' Lambda	,099	168,001[b]	2,000	37,000	<,001
		Hotelling's Trace	9,081	168,001[b]	2,000	37,000	<,001
		Roy's Largest Root	9,081	168,001[b]	2,000	37,000	<,001
	Gruppe	Pillai's Trace	,080	1,616[b]	2,000	37,000	,212
		Wilks' Lambda	,920	1,616[b]	2,000	37,000	,212
		Hotelling's Trace	,087	1,616[b]	2,000	37,000	,212
		Roy's Largest Root	,087	1,616[b]	2,000	37,000	,212

a. Design: Intercept + Gruppe

b. Exact statistic

Tests of Between-Subjects Effects

age_category	Source	Dependent Variable	Type III Sum of Squares	df	Mean Square	F	Sig.
.	Corrected Model	MADRS_discharge	66,667[a]	1	66,667	,926	,512
		MittelSchritte_discharge	15477022,04[b]	1	15477022,042	,576	,587
	Intercept	MADRS_discharge	682,667	1	682,667	9,481	,200
		MittelSchritte_discharge	68374128,375	1	68374128,375	2,543	,357
	Gruppe	MADRS_discharge	66,667	1	66,667	,926	,512
		MittelSchritte_discharge	15477022,042	1	15477022,042	,576	,587
	Error	MADRS_discharge	72,000	1	72,000		
		MittelSchritte_discharge	26882778,125	1	26882778,125		
	Total	MADRS_discharge	755,000	3			
		MittelSchritte_discharge	145613133,50	3			
	Corrected Total	MADRS_discharge	138,667	2			
		MittelSchritte_discharge	42359800,167	2			
younger than 44	Corrected Model	MADRS_discharge	,833[c]	1	,833	,008	,930
		MittelSchritte_discharge	439944,190[d]	1	439944,190	,043	,836
	Intercept	MADRS_discharge	13696,033	1	13696,033	129,181	<,001
		MittelSchritte_discharge	2130217234,0	1	2130217234,0	210,292	<,001
	Gruppe	MADRS_discharge	,833	1	,833	,008	,930
		MittelSchritte_discharge	439944,190	1	439944,190	,043	,836
	Error	MADRS_discharge	4558,944	43	106,022		
		MittelSchritte_discharge	435581370,42	43	10129799,312		
	Total	MADRS_discharge	18782,000	45			
		MittelSchritte_discharge	2667771490,1	45			
	Corrected Total	MADRS_discharge	4559,778	44			
		MittelSchritte_discharge	436021314,61	44			
older than 43	Corrected Model	MADRS_discharge	348,100[e]	1	348,100	3,319	,076
		MittelSchritte_discharge	406069,518[f]	1	406069,518	,037	,848
	Intercept	MADRS_discharge	10562,500	1	10562,500	100,711	<,001
		MittelSchritte_discharge	2294135339,4	1	2294135339,4	211,328	<,001
	Gruppe	MADRS_discharge	348,100	1	348,100	3,319	,076
		MittelSchritte_discharge	406069,518	1	406069,518	,037	,848
	Error	MADRS_discharge	3985,400	38	104,879		
		MittelSchritte_discharge	412521242,03	38	10855822,159		
	Total	MADRS_discharge	14896,000	40			
		MittelSchritte_discharge	2707062650,9	40			
	Corrected Total	MADRS_discharge	4333,500	39			
		MittelSchritte_discharge	412927311,55	39			

a. R Squared = ,481 (Adjusted R Squared = -,038)

b. R Squared = ,365 (Adjusted R Squared = -,269)

c. R Squared = ,000 (Adjusted R Squared = -,023)

d. R Squared = ,001 (Adjusted R Squared = -,022)

e. R Squared = ,080 (Adjusted R Squared = ,056)

f. R Squared = ,001 (Adjusted R Squared = -,025)

S8c Subgroup analysis for gender

Multivariate Tests[a]

Geschlecht T0	Effect		Value	F	Hypothesis df	Error df	Sig.
no answer	Intercept	Pillai's Trace	.	.[b]	.	.	.
		Wilks' Lambda	.	.[b]	.	.	.
		Hotelling's Trace	.	.[b]	.	.	.
		Roy's Largest Root	.	.[b]	.	.	.
	Gruppe	Pillai's Trace	.	.[b]	.	.	.
		Wilks' Lambda	.	.[b]	.	.	.
		Hotelling's Trace	.	.[b]	.	.	.
		Roy's Largest Root	.	.[b]	.	.	.
female	Intercept	Pillai's Trace	,912	247,599[b]	2,000	48,000	<,001
		Wilks' Lambda	,088	247,599[b]	2,000	48,000	<,001
		Hotelling's Trace	10,317	247,599[b]	2,000	48,000	<,001
		Roy's Largest Root	10,317	247,599[b]	2,000	48,000	<,001
	Gruppe	Pillai's Trace	,082	2,158[b]	2,000	48,000	,127
		Wilks' Lambda	,918	2,158[b]	2,000	48,000	,127
		Hotelling's Trace	,090	2,158[b]	2,000	48,000	,127
		Roy's Largest Root	,090	2,158[b]	2,000	48,000	,127
male	Intercept	Pillai's Trace	,886	124,827[b]	2,000	32,000	<,001
		Wilks' Lambda	,114	124,827[b]	2,000	32,000	<,001
		Hotelling's Trace	7,802	124,827[b]	2,000	32,000	<,001
		Roy's Largest Root	7,802	124,827[b]	2,000	32,000	<,001
	Gruppe	Pillai's Trace	,002	,034[b]	2,000	32,000	,967
		Wilks' Lambda	,998	,034[b]	2,000	32,000	,967
		Hotelling's Trace	,002	,034[b]	2,000	32,000	,967
		Roy's Largest Root	,002	,034[b]	2,000	32,000	,967
divers	Intercept	Pillai's Trace	.	.[b]	.	.	.
		Wilks' Lambda	.	.[b]	.	.	.
		Hotelling's Trace	.	.[b]	.	.	.
		Roy's Largest Root	.	.[b]	.	.	.
	Gruppe	Pillai's Trace	.	.[b]	.	.	.
		Wilks' Lambda	.	.[b]	.	.	.
		Hotelling's Trace	.	.[b]	.	.	.
		Roy's Largest Root	.	.[b]	.	.	.

a. Design: Intercept + Gruppe

b. Exact statistic

Tests of Between-Subjects Effects

Geschlecht T0	Source	Dependent Variable	Type III Sum of Squares	df	Mean Square	F	Sig.
no answer	Corrected Model	MADRS_discharge	,000[a]	0	.	.	.
		MittelSchritte_discharge	,000[a]	0	.	.	.
	Intercept	MADRS_discharge	25,000	1	25,000	.	.
		MittelSchritte_discharge	14489442,250	1	14489442,250	.	.
	Gruppe	MADRS_discharge	,000	0	.	.	.
		MittelSchritte_discharge	,000	0	.	.	.
	Error	MADRS_discharge	,000	0	.		
		MittelSchritte_discharge	,000	0	.		
	Total	MADRS_discharge	25,000	1			
		MittelSchritte_discharge	14489442,250	1			
	Corrected Total	MADRS_discharge	,000	0			
		MittelSchritte_discharge	,000	0			
female	Corrected Model	MADRS_discharge	449,460[b]	1	449,460	4,118	,048
		MittelSchritte_discharge	242781,510[c]	1	242781,510	,023	,879
	Intercept	MADRS_discharge	15736,519	1	15736,519	144,170	<,001
		MittelSchritte_discharge	2831441398,5	1	2831441398,5	271,124	<,001
	Gruppe	MADRS_discharge	449,460	1	449,460	4,118	,048
		MittelSchritte_discharge	242781,510	1	242781,510	,023	,879
	Error	MADRS_discharge	5348,462	49	109,152		
		MittelSchritte_discharge	511723200,99	49	10443330,632		
	Total	MADRS_discharge	21645,000	51			
		MittelSchritte_discharge	3345525071,1	51			
	Corrected Total	MADRS_discharge	5797,922	50			
		MittelSchritte_discharge	511965982,50	50			
male	Corrected Model	MADRS_discharge	5,564[d]	1	5,564	,063	,803
		MittelSchritte_discharge	94205,968[e]	1	94205,968	,008	,927
	Intercept	MADRS_discharge	8418,821	1	8418,821	95,790	<,001
		MittelSchritte_discharge	1647059141,7	1	1647059141,7	147,240	<,001
	Gruppe	MADRS_discharge	5,564	1	5,564	,063	,803
		MittelSchritte_discharge	94205,968	1	94205,968	,008	,927
	Error	MADRS_discharge	2900,322	33	87,889		
		MittelSchritte_discharge	369146358,68	33	11186253,293		
	Total	MADRS_discharge	11802,000	35			
		MittelSchritte_discharge	2139784825,1	35			
	Corrected Total	MADRS_discharge	2905,886	34			
		MittelSchritte_discharge	369240564,64	34			
divers	Corrected Model	MADRS_discharge	,000[a]	0	.	.	.
		MittelSchritte_discharge	,000[a]	0	.	.	.
	Intercept	MADRS_discharge	961,000	1	961,000	.	.
		MittelSchritte_discharge	20647936,000	1	20647936,000	.	.
	Gruppe	MADRS_discharge	,000	0	.	.	.
		MittelSchritte_discharge	,000	0	.	.	.
	Error	MADRS_discharge	,000	0	.		
		MittelSchritte_discharge	,000	0	.		
	Total	MADRS_discharge	961,000	1			
		MittelSchritte_discharge	20647936,000	1			
	Corrected Total	MADRS_discharge	,000	0			
		MittelSchritte_discharge	,000	0			

a. R Squared = . (Adjusted R Squared = .)

b. R Squared = ,078 (Adjusted R Squared = ,059)

c. R Squared = ,000 (Adjusted R Squared = -,020)

d. R Squared = ,002 (Adjusted R Squared = -,028)

e. R Squared = ,000 (Adjusted R Squared = -,030)

S8d Subgroup analysis for responders versus non-responders

Tests of Between-Subjects Effects

MADRS_response_50Kategorie	Source	Dependent Variable	Type III Sum of Squares	df	Mean Square	F	Sig.
non-responder	Corrected Model	MADRS_discharge	16,850[a]	1	16,850	,310	,580
		MittelSchritte_discharge	23491346,13[b]	1	23491346,127	3,117	,084
	Intercept	MADRS_discharge	27720,360	1	27720,360	509,813	<,001
		MittelSchritte_discharge	2235001141,6	1	2235001141,6	296,543	<,001
	Gruppe	MADRS_discharge	16,850	1	16,850	,310	,580
		MittelSchritte_discharge	23491346,127	1	23491346,127	3,117	,084
	MADRS_response50Kategorie	MADRS_discharge	,000	0	.	.	.
		MittelSchritte_discharge	,000	0	.	.	.
	Gruppe * MADRS_response50Kategorie	MADRS_discharge	,000	0	.	.	.
		MittelSchritte_discharge	,000	0	.	.	.
	Error	MADRS_discharge	2555,559	47	54,374		
		MittelSchritte_discharge	354231553,00	47	7536841,553		
	Total	MADRS_discharge	31521,000	49			
		MittelSchritte_discharge	2778687665,9	49			
	Corrected Total	MADRS_discharge	2572,408	48			
		MittelSchritte_discharge	377722899,12	48			
responder	Corrected Model	MADRS_discharge	4,447[c]	1	4,447	,296	,590
		MittelSchritte_discharge	17500392,81[d]	1	17500392,811	1,268	,268
	Intercept	MADRS_discharge	2197,921	1	2197,921	146,357	<,001
		MittelSchritte_discharge	2197863506,5	1	2197863506,5	159,262	<,001
	Gruppe	MADRS_discharge	4,447	1	4,447	,296	,590
		MittelSchritte_discharge	17500392,811	1	17500392,811	1,268	,268
	MADRS_response50Kategorie	MADRS_discharge	,000	0	.	.	.
		MittelSchritte_discharge	,000	0	.	.	.
	Gruppe * MADRS_response50Kategorie	MADRS_discharge	,000	0	.	.	.
		MittelSchritte_discharge	,000	0	.	.	.
	Error	MADRS_discharge	540,632	36	15,018		
		MittelSchritte_discharge	496809362,17	36	13800260,060		
	Total	MADRS_discharge	2743,000	38			
		MittelSchritte_discharge	2712173261,5	38			
	Corrected Total	MADRS_discharge	545,079	37			
		MittelSchritte_discharge	514309754,99	37			

a. R Squared = ,007 (Adjusted R Squared = -,015)

b. R Squared = ,062 (Adjusted R Squared = ,042)

c. R Squared = ,008 (Adjusted R Squared = -,019)

d. R Squared = ,034 (Adjusted R Squared = ,007)

Multivariate Tests[a]

MADRS_response_50Kategorie	Effect		Value	F	Hypothesis df	Error df	Sig.
non-responder	Intercept	Pillai's Trace	,957	507,288[b]	2,000	46,000	<,001
		Wilks' Lambda	,043	507,288[b]	2,000	46,000	<,001
		Hotelling's Trace	22,056	507,288[b]	2,000	46,000	<,001
		Roy's Largest Root	22,056	507,288[b]	2,000	46,000	<,001
	Gruppe	Pillai's Trace	,080	1,999[b]	2,000	46,000	,147
		Wilks' Lambda	,920	1,999[b]	2,000	46,000	,147
		Hotelling's Trace	,087	1,999[b]	2,000	46,000	,147
		Roy's Largest Root	,087	1,999[b]	2,000	46,000	,147
	MADRS_response50Kategorie	Pillai's Trace	,000	.[b]	,000	,000	.
		Wilks' Lambda	1,000	.[b]	,000	46,500	.
		Hotelling's Trace	,000	.[b]	,000	2,000	.
		Roy's Largest Root	,000	,000[b]	2,000	45,000	1,000
	Gruppe * MADRS_response50Kategorie	Pillai's Trace	,000	.[b]	,000	,000	.
		Wilks' Lambda	1,000	.[b]	,000	46,500	.
		Hotelling's Trace	,000	.[b]	,000	2,000	.
		Roy's Largest Root	,000	,000[b]	2,000	45,000	1,000
responder	Intercept	Pillai's Trace	,881	129,929[b]	2,000	35,000	<,001
		Wilks' Lambda	,119	129,929[b]	2,000	35,000	<,001
		Hotelling's Trace	7,424	129,929[b]	2,000	35,000	<,001
		Roy's Largest Root	7,424	129,929[b]	2,000	35,000	<,001
	Gruppe	Pillai's Trace	,047	,864[b]	2,000	35,000	,430
		Wilks' Lambda	,953	,864[b]	2,000	35,000	,430
		Hotelling's Trace	,049	,864[b]	2,000	35,000	,430
		Roy's Largest Root	,049	,864[b]	2,000	35,000	,430
	MADRS_response50Kategorie	Pillai's Trace	,000	.[b]	,000	,000	.
		Wilks' Lambda	1,000	.[b]	,000	35,500	.
		Hotelling's Trace	,000	.[b]	,000	2,000	.
		Roy's Largest Root	,000	,000[b]	2,000	34,000	1,000
	Gruppe * MADRS_response50Kategorie	Pillai's Trace	,000	.[b]	,000	,000	.
		Wilks' Lambda	1,000	.[b]	,000	35,500	.
		Hotelling's Trace	,000	.[b]	,000	2,000	.
		Roy's Largest Root	,000	,000[b]	2,000	34,000	1,000

a. Design: Intercept + Gruppe + MADRS_response50Kategorie + Gruppe * MADRS_response50Kategorie

b. Exact statistic

S8e Correlations between steps and MADRS for moderate and severe depression

Correlations

MADRS_severity			steps per day baseline	MADRS score baseline
moderate	steps per day baseline	Pearson Correlation	1	-,088
		Sig. (2-tailed)		,417
		N	87	87
	MADRS score baseline	Pearson Correlation	-,088	1
		Sig. (2-tailed)	,417	
		N	87	132
severe	steps per day baseline	Pearson Correlation	1	-,606**
		Sig. (2-tailed)		<,001
		N	27	27
	MADRS score baseline	Pearson Correlation	-,606**	1
		Sig. (2-tailed)	<,001	
		N	27	44

**. Correlation is significant at the 0.01 level (2-tailed).

8f: Physical activity on different weekdays

Weekday	N	Mean	Std. Deviation
**Monday**	*18*	*5678,71*	*2892,30*
**Tuesday**	*20*	*7269,32*	*2389,23*
**Wednesday**	*42*	*6685,79*	*3175,35*
**Thursday**	*65*	*6568,61*	*2646,72*
**Friday**	*74*	*6326,51*	*3343,85*
**Saturday**	*63*	*6800,33*	*3204,88*
**Sunday**	*45*	*5375,92*	*2617,95*

Steps per day on different weekdays at baseline

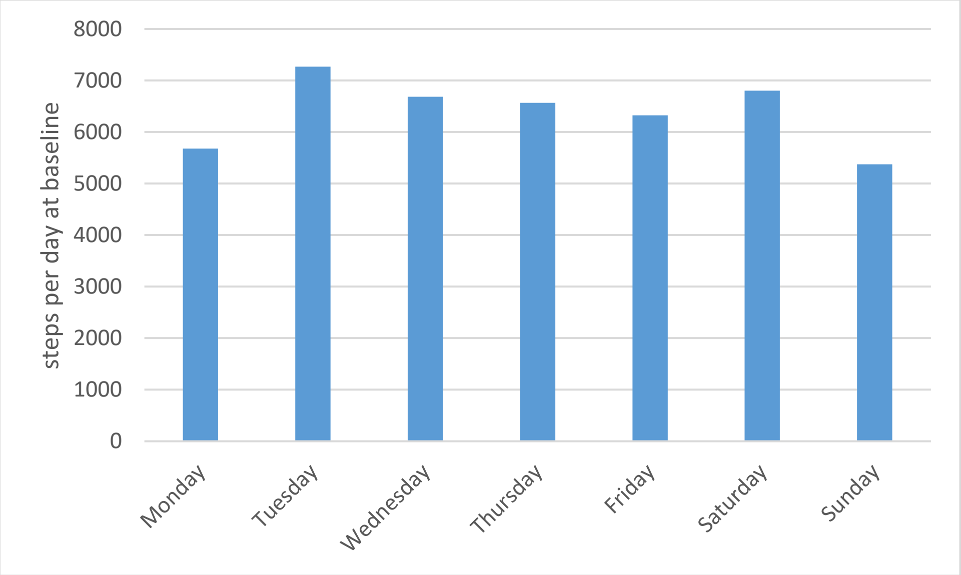

Averaged steps per day at baseline for single weekdays

S9: Program evaluation ratings

Descriptive Statistics

		Evaluation:	Activity	Conscientousness	Pedometer	Note
Question		How helpful was the activity program for you?	Did the activity program help you to be more physically active in your daily life?	How carefully did you pursue your current step goal in the last week?	Did you pay more attention to your physical activity due to the pedometer?	Which note do you give the activity program altogether?
Values		1 = not helpful at all *To* 6 = very helpful	1 = not helpful at all *To* 6 = very helpful	1 = not carefully at all *To* 6 = very carefully	1 = not at all *To* 6 = very much	1 = excellent 2 = good 3 = satisfactory 4 = sufficient 5 = insufficient 6 = fail
N	Valid	69	69	68	68	67
	Missing	123	123	124	124	125
Mean		4,07	4,06	3,60	3,94	2,16
Median		4,00	4,00	4,00	4,00	2,00
Std. Deviation		1,343	1,514	1,658	1,769	,931
Range		5	5	5	5	4
Minimum		1	1	1	1	1
Maximum		6	6	6	6	5

S10: Program evaluation – categories for free answers

N = 35		
What did you like about this program?		
13	37,10%	it was motivating
8	22,90%	the monitoring
4	11,40%	having more control about my physical activity
3	8,60%	participation in the study/communication
2	5,70%	the concept of the study
2	5,70%	the offer of the study
2	5,70%	having moved more
1	2,90%	simplicity
1	2,90%	success
1	2,90%	structure of the program
1	2,90%	it was not motivating
What did you not like about the program?		
5	14,3%	the accelerometer measure
4	11,4%	nothing
3	8,6%	too simple
2	5,7%	not motivating
2	5,7%	measures
2	5,7%	questions of the IPAQ
2	5,7%	too less support
1	2,9%	depressive although walking
1	2,9%	too much physical activity
1	2,9%	the pedometer
1	2,9%	500 steps are too many per week
What could be made better?		
3	8,6%	an arm wrist tool
2	5,7%	more support
1	2,9%	reward
1	2,9%	to include other types of physical activity
1	2,9%	the instruction not to move more at baseline
1	2,9%	digital format
1	2,9%	mor offers to be physically active
1	2,9%	more destinations to walk to
